# 2,2,2-Tribromo-*N*-(3-chloro­phen­yl)acetamide

**DOI:** 10.1107/S160053681001411X

**Published:** 2010-04-24

**Authors:** P. A. Suchetan, B. Thimme Gowda, Sabine Foro, Hartmut Fuess

**Affiliations:** a Department of Chemistry, Mangalore University, Mangalagangotri 574 199, Mangalore, India; b Institute of Materials Science, Darmstadt University of Technology, Petersenstrasse 23, D-64287 Darmstadt, Germany

## Abstract

In the title compound, C_8_H_5_Br_3_ClNO, the conformation of the N—H bond is *anti* to the 3-chloro substituent in the benzene ring. An intra­molecular N—H⋯Br hydrogen bond occurs. In the crystal, mol­ecules are packed into infinite chains in the *a-*axis direction by N—H⋯O hydrogen bonds.

## Related literature

For the preparation of the title compound, see: Gowda *et al.* (2003 ▶). For background and related structures, see: Brown (1966 ▶); Gowda *et al.* (2008 ▶, 2009 ▶, 2010 ▶).
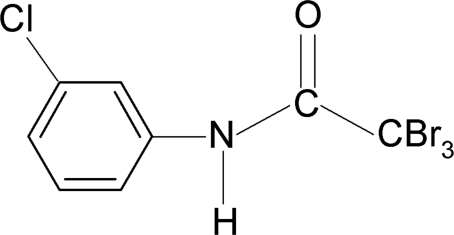


## Experimental

### 

#### Crystal data

C_8_H_5_Br_3_ClNO
*M* *_r_* = 406.31
Orthorhombic, 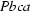

*a* = 12.803 (1) Å
*b* = 9.146 (1) Å
*c* = 20.221 (3) Å
*V* = 2367.8 (5) Å^3^
*Z* = 8
Cu *K*α radiation
μ = 14.47 mm^−1^
*T* = 299 K
0.53 × 0.33 × 0.25 mm

#### Data collection

Enraf–Nonius CAD-4 diffractometer
Absorption correction: ψ scan (North *et al.*, 1968 ▶) *T*_min_ = 0.049, *T*_max_ = 0.123
3870 measured reflections
2114 independent reflections
1646 reflections with *I* > 2σ(*I*)
*R*_int_ = 0.110
3 standard reflections every 120 min  intensity decay: 1.5%

#### Refinement

*R*[*F* ^2^ > 2σ(*F* ^2^)] = 0.086
*wR*(*F* ^2^) = 0.387
*S* = 1.59
2114 reflections
127 parameters
H-atom parameters constrained
Δρ_max_ = 2.07 e Å^−3^
Δρ_min_ = −1.56 e Å^−3^

### 

Data collection: *CAD-4-PC* (Enraf–Nonius, 1996 ▶); cell refinement: *CAD-4-PC*; data reduction: *REDU4* (Stoe & Cie, 1987 ▶); program(s) used to solve structure: *SHELXS97* (Sheldrick, 2008 ▶); program(s) used to refine structure: *SHELXL97* (Sheldrick, 2008 ▶); molecular graphics: *PLATON* (Spek, 2009 ▶); software used to prepare material for publication: *SHELXL97*.

## Supplementary Material

Crystal structure: contains datablocks I, global. DOI: 10.1107/S160053681001411X/fl2301sup1.cif

Structure factors: contains datablocks I. DOI: 10.1107/S160053681001411X/fl2301Isup2.hkl

Additional supplementary materials:  crystallographic information; 3D view; checkCIF report

## Figures and Tables

**Table 1:** Hydrogen-bond geometry (Å, °)

*D*—H⋯*A*	*D*—H	H⋯*A*	*D*⋯*A*	*D*—H⋯*A*
N1—H1*N*⋯O1^i^	0.86	2.20	3.032 (13)	162
N1—H1*N*⋯Br3	0.86	2.84	3.177 (9)	105

Symmetry code: (i) 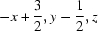.

**Fig. 1.**
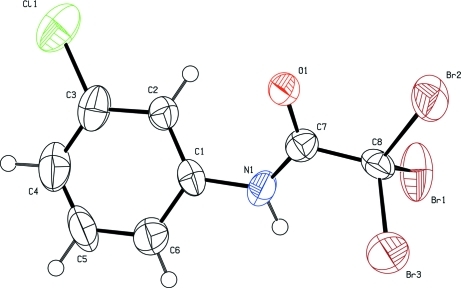
Molecular structure of (I), showing the atom labelling scheme. Displacement ellipsoids are drawn at the 50% probability level and H atoms are represented as small spheres of arbitrary radii.

**Fig. 2.**
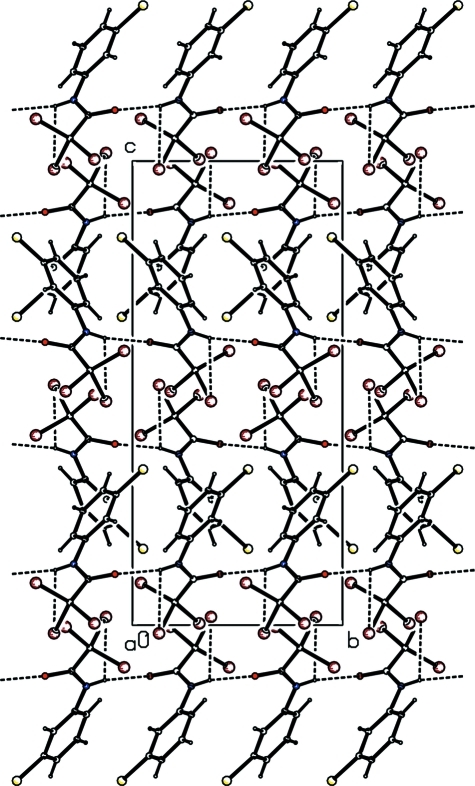
Molecular packing of (I) with hydrogen bonds shown as dashed lines.

## supplementary crystallographic information

### Comment

The structure of (I), was determined as a part of our ongoing study of the effect of  ring and side chain substituents on the crystal structures of *N*-aromatic amides (Gowda *et al.*, 2008, 2009, 2010). In (I) the conformation of the N—H bond is *anti* to the 3-chloro substituent in the benzene ring (Fig.1), similar to that observed in *N*-(3-chlorophenyl)acetamide (II)(Gowda *et al.*, 2008), and that between the N—H bond and the 3-methyl group in *N*-(3-methylphenyl)2,2,2-tribromoacetamide (III)(Gowda *et al.*, 2009), but contrary to the *syn* conformation observed between the N—H bond and the 2-Chloro group in *N*-(2-chlorophenyl)2,2,2-tribromoacetamide (IV) (Gowda *et al.*, 2010).

Further, the conformation of the N—H bond in (I) is *anti* to the C=O bond in the side chain, similar to that observed in *N*-(phenyl)2,2,2-tribromoacetamide, (II), (III) and (IV) (Gowda *et al.*, 2008, 2009, 2010) and other amides (Brown, 1966).

The structure of (I) shows both  intramolecular N—H···Br and intermolecular N—H···O H-bonding. A packing diagram (Fig. 2)  illustrates the N1—H1N···O1 hydrogen bonds  (Table 1) involved in the formation of molecular chains along the *a*-axis of the unit cell.

### Experimental

The title compound was prepared from 3-chloroaniline, tribromoacetic acid and phosphorylchloride according to the literature method (Gowda *et al.*, 2003). The purity of the compound was checked by determining its melting point. It was further characterized by recording its infrared spectra. Rod like colourless single crystals of the title compound used for X-ray diffraction studies were obtained by a slow evaporation of its ethanolic solution at room temperature.

### Refinement

The H atoms were positioned with idealized geometry using a riding model [N—H = 0.86 Å, C—H = 0.93 Å] and were refined with a riding model conith isotropic displacement parameters (set to 1.2 times of the U_eq_ of the parent atom).

The residual electron-density features are located in the region of Br1 and Br2. The highest peak is 0.98 Å from Br1 and the deepest hole is 1.39 Å from Br2.

### Crystal data

**Table:**

C_8_H_5_Br_3_ClNO	*F*(000) = 1520
*M**_r_* = 406.31	*D*_x_ = 2.280 Mg m^−^^3^
Orthorhombic, *P**b**c**a*	Cu *K*α radiation, λ = 1.54180 Å
Hall symbol: -P 2ac 2ab	Cell parameters from 25 reflections
*a* = 12.803 (1) Å	θ = 4.4–20.5°
*b* = 9.146 (1) Å	µ = 14.47 mm^−^^1^
*c* = 20.221 (3) Å	*T* = 299 K
*V* = 2367.8 (5) Å^3^	Rod, colourless
*Z* = 8	0.53 × 0.33 × 0.25 mm

### Data collection

**Table:**

Enraf–Nonius CAD-4 diffractometer	1646 reflections with *I* > 2σ(*I*)
Radiation source: fine-focus sealed tube	*R*_int_ = 0.110
graphite	θ_max_ = 67.0°, θ_min_ = 4.4°
ω/2θ scans	*h* = −15→11
Absorption correction: ψ scan (North *et al.*, 1968)	*k* = −10→0
*T*_min_ = 0.049, *T*_max_ = 0.123	*l* = −24→0
3870 measured reflections	3 standard reflections every 120 min
2114 independent reflections	intensity decay: 1.5%

### Refinement

**Table:**

Refinement on *F*^2^	Primary atom site location: structure-invariant direct methods
Least-squares matrix: full	Secondary atom site location: difference Fourier map
*R*[*F*^2^ > 2σ(*F*^2^)] = 0.086	Hydrogen site location: inferred from neighbouring sites
*wR*(*F*^2^) = 0.387	H-atom parameters constrained
*S* = 1.59	*w* = 1/[σ^2^(*F*_o_^2^) + (0.2*P*)^2^] where *P* = (*F*_o_^2^ + 2*F*_c_^2^)/3
2114 reflections	(Δ/σ)_max_ = 0.006
127 parameters	Δρ_max_ = 2.07 e Å^−^^3^
0 restraints	Δρ_min_ = −1.56 e Å^−^^3^

### Special details

**Table:**

Geometry. All esds (except the esd in the dihedral angle between two l.s. planes) are estimated using the full covariance matrix. The cell esds are taken into account individually in the estimation of esds in distances, angles and torsion angles; correlations between esds in cell parameters are only used when they are defined by crystal symmetry. An approximate (isotropic) treatment of cell esds is used for estimating esds involving l.s. planes.
Refinement. Refinement of *F*^2^ against ALL reflections. The weighted *R*-factor wR and goodness of fit *S* are based on *F*^2^, conventional *R*-factors *R* are based on *F*, with *F* set to zero for negative *F*^2^. The threshold expression of *F*^2^ > σ(*F*^2^) is used only for calculating *R*-factors(gt) etc. and is not relevant to the choice of reflections for refinement. *R*-factors based on *F*^2^ are statistically about twice as large as those based on *F*, and *R*- factors based on ALL data will be even larger.

### Fractional atomic coordinates and isotropic or equivalent isotropic displacement parameters (Å^2^)

**Table:**

	*x*	*y*	*z*	*U*_iso_*/*U*_eq_	
C1	0.8142 (8)	0.2739 (12)	0.3141 (5)	0.042 (2)	
C2	0.7763 (8)	0.3768 (12)	0.2709 (6)	0.043 (2)	
H2	0.7101	0.4167	0.2769	0.051*	
C3	0.8383 (13)	0.4204 (15)	0.2184 (6)	0.057 (3)	
C4	0.9361 (13)	0.3617 (16)	0.2099 (8)	0.071 (4)	
H4	0.9773	0.3902	0.1743	0.085*	
C5	0.9715 (11)	0.2635 (18)	0.2533 (9)	0.077 (5)	
H5	1.0374	0.2231	0.2467	0.092*	
C6	0.9141 (11)	0.2199 (13)	0.3074 (8)	0.059 (3)	
H6	0.9419	0.1559	0.3385	0.071*	
C7	0.6833 (9)	0.2898 (11)	0.4008 (5)	0.042 (2)	
C8	0.6221 (8)	0.2020 (11)	0.4511 (6)	0.043 (2)	
Br1	0.55384 (15)	0.04007 (19)	0.40907 (9)	0.0787 (8)	
Br2	0.52069 (17)	0.31822 (18)	0.49631 (11)	0.0893 (9)	
Br3	0.71709 (15)	0.1263 (3)	0.51772 (8)	0.0856 (8)	
Cl1	0.7921 (4)	0.5505 (5)	0.16439 (19)	0.0817 (13)	
N1	0.7560 (8)	0.2201 (11)	0.3684 (5)	0.050 (2)	
H1N	0.7701	0.1327	0.3814	0.060*	
O1	0.6555 (7)	0.4164 (8)	0.3902 (4)	0.0494 (19)	

### Atomic displacement parameters (Å^2^)

**Table:**

	*U*^11^	*U*^22^	*U*^33^	*U*^12^	*U*^13^	*U*^23^
C1	0.032 (5)	0.046 (5)	0.047 (6)	−0.008 (4)	0.008 (4)	−0.005 (5)
C2	0.036 (5)	0.045 (5)	0.048 (6)	−0.006 (4)	0.000 (4)	0.005 (4)
C3	0.069 (8)	0.065 (7)	0.039 (5)	−0.018 (6)	0.006 (5)	0.001 (5)
C4	0.082 (10)	0.051 (7)	0.079 (10)	−0.009 (7)	0.041 (8)	−0.003 (7)
C5	0.045 (8)	0.070 (9)	0.116 (12)	−0.003 (7)	0.040 (8)	0.004 (10)
C6	0.060 (7)	0.040 (5)	0.076 (8)	0.003 (5)	0.017 (6)	0.004 (6)
C7	0.044 (6)	0.037 (5)	0.044 (5)	−0.002 (4)	−0.001 (4)	0.000 (4)
C8	0.032 (5)	0.037 (5)	0.061 (6)	0.006 (4)	0.004 (5)	−0.005 (4)
Br1	0.0826 (13)	0.0758 (12)	0.0776 (12)	−0.0431 (9)	0.0248 (8)	−0.0149 (8)
Br2	0.0953 (15)	0.0610 (12)	0.1114 (16)	0.0226 (9)	0.0628 (12)	0.0087 (9)
Br3	0.0700 (12)	0.1248 (18)	0.0619 (12)	0.0134 (10)	−0.0027 (7)	0.0327 (10)
Cl1	0.117 (3)	0.079 (2)	0.0498 (19)	−0.006 (2)	0.0019 (18)	0.0161 (16)
N1	0.056 (6)	0.041 (4)	0.052 (5)	0.000 (4)	0.016 (5)	0.015 (4)
O1	0.042 (4)	0.037 (3)	0.069 (5)	0.000 (3)	0.008 (4)	0.009 (3)

### Geometric parameters (Å, °)

**Table:**

C1—C2	1.372 (16)	C5—H5	0.9300
C1—C6	1.378 (17)	C6—H6	0.9300
C1—N1	1.416 (13)	C7—O1	1.229 (14)
C2—C3	1.385 (16)	C7—N1	1.305 (16)
C2—H2	0.9300	C7—C8	1.515 (15)
C3—C4	1.37 (2)	C8—Br2	1.911 (10)
C3—Cl1	1.720 (15)	C8—Br1	1.918 (11)
C4—C5	1.33 (2)	C8—Br3	1.943 (11)
C4—H4	0.9300	N1—H1N	0.8600
C5—C6	1.377 (18)		
			
C2—C1—C6	120.8 (10)	C5—C6—C1	118.0 (14)
C2—C1—N1	123.1 (10)	C5—C6—H6	121.0
C6—C1—N1	116.1 (11)	C1—C6—H6	121.0
C1—C2—C3	118.8 (11)	O1—C7—N1	125.4 (10)
C1—C2—H2	120.6	O1—C7—C8	117.8 (10)
C3—C2—H2	120.6	N1—C7—C8	116.5 (9)
C4—C3—C2	120.4 (13)	C7—C8—Br2	112.2 (7)
C4—C3—Cl1	120.3 (10)	C7—C8—Br1	110.4 (8)
C2—C3—Cl1	119.3 (12)	Br2—C8—Br1	109.4 (5)
C5—C4—C3	119.4 (12)	C7—C8—Br3	109.3 (7)
C5—C4—H4	120.3	Br2—C8—Br3	107.0 (6)
C3—C4—H4	120.3	Br1—C8—Br3	108.5 (5)
C4—C5—C6	122.4 (14)	C7—N1—C1	126.5 (10)
C4—C5—H5	118.8	C7—N1—H1N	116.8
C6—C5—H5	118.8	C1—N1—H1N	116.8
			
C6—C1—C2—C3	3.2 (17)	O1—C7—C8—Br2	−6.9 (13)
N1—C1—C2—C3	−178.6 (11)	N1—C7—C8—Br2	178.6 (9)
C1—C2—C3—C4	−0.3 (19)	O1—C7—C8—Br1	115.4 (10)
C1—C2—C3—Cl1	−179.4 (9)	N1—C7—C8—Br1	−59.1 (12)
C2—C3—C4—C5	−1(2)	O1—C7—C8—Br3	−125.4 (9)
Cl1—C3—C4—C5	178.3 (13)	N1—C7—C8—Br3	60.1 (12)
C3—C4—C5—C6	−1(3)	O1—C7—N1—C1	−2(2)
C4—C5—C6—C1	4(2)	C8—C7—N1—C1	171.9 (11)
C2—C1—C6—C5	−5(2)	C2—C1—N1—C7	−28.2 (19)
N1—C1—C6—C5	176.7 (13)	C6—C1—N1—C7	150.0 (13)

### Hydrogen-bond geometry (Å, °)

**Table:**

*D*—H···*A*	*D*—H	H···*A*	*D*···*A*	*D*—H···*A*
N1—H1N···O1^i^	0.86	2.20	3.032 (13)	162
N1—H1N···Br3	0.86	2.84	3.177 (9)	105

Symmetry codes: (i) −*x*+3/2, *y*−1/2, *z*.
